# Discovery of low-molecular weight anti-PD-L1 peptides for cancer immunotherapy

**DOI:** 10.1186/s40425-019-0705-y

**Published:** 2019-10-22

**Authors:** Hao Liu, Zhen Zhao, Li Zhang, Yuanke Li, Akshay Jain, Ashutosh Barve, Wei Jin, Yanli Liu, John Fetse, Kun Cheng

**Affiliations:** Division of Pharmacology and Pharmaceutical Sciences, School of Pharmacy, University of Missouri-Kansas City, 2464 Charlotte Street, Kansas City, MO 64108 USA

**Keywords:** Peptide, Checkpoint inhibitor, PD-L1, PD-1, Phage display, Tumor penetration, CT26

## Abstract

**Background:**

Immunotherapy using checkpoint inhibitors, especially PD-1/PD-L1 inhibitors, has now evolved into the most promising therapy for cancer patients. However, most of these inhibitors are monoclonal antibodies, and their large size may limit their tumor penetration, leading to suboptimal efficacy. As a result, there has been a growing interest in developing low-molecular-weight checkpoint inhibitors.

**Methods:**

We developed a novel biopanning strategy to discover small peptide-based anti-PD-L1 inhibitors. The affinity and specificity of the peptides to PD-L1 were examined using various assays. Three-dimensional (3D) spheroid penetration study was performed to determine the tumor penetration capability of the peptides. Anti-tumor activity of the peptides was evaluated in mice bearing CT26 tumor cells.

**Results:**

We discover several anti-PD-L1 peptide inhibitors to block PD-1/PD-L1 interaction. The peptides exhibit high affinity and specificity to human PD-L1 protein as well as PD-L1-overexpressing human cancer cells MDA-MB-231 and DU-145. Molecular docking studies indicate that the peptide CLP002 specifically binds to PD-L1 at the residues where PD-L1 interacts with PD-1. The peptide also blocks the CD80/PD-L1 interaction, which may further enhance the immune response of tumor-infiltrating T cells. Compared to antibody, the peptide CLP002 exhibits better tumor penetration in a 3D tumor spheroid model. The peptide CLP002 restores proliferation and prevents apoptosis of T cells that are co-cultured with cancer cells. The peptide CLP002 also inhibits tumor growth and increases survival of CT26 tumor-bearing mice.

**Conclusions:**

This study demonstrated the feasibility of using phage display to discover small peptide-based checkpoint inhibitors. Our results also suggested that the anti-PD-L1 peptide represents a promising low-molecular-weight checkpoint inhibitor for cancer immunotherapy.

**Electronic supplementary material:**

The online version of this article (10.1186/s40425-019-0705-y) contains supplementary material, which is available to authorized users.

## Background

Immunotherapy using checkpoint inhibitors has now evolved into the most promising cancer therapy with remarkable responses. Checkpoint inhibitors modulate the tumor cell-immune cell interaction and subsequently prompt the patient’s own immune system to destroy tumor cells. Among the multiple checkpoint inhibitors, the programmed death-1 (PD-1)/programmed death-ligand 1 (PD-L1) inhibitors have achieved the most brilliant success in clinical applications [1, 2]. PD-L1 is overexpressed in various cancer cells, and the binding of PD-L1 to PD-1, which is expressed on immune cells, leads to immunosuppressive activity of T cells. Blockade of the PD-1/PD-L1 interaction therefore disrupts the immune-suppressing pathway and unleashes the anti-cancer immune responses of the T cells to destroy cancer cells [1, 2]. Three PD-L1 inhibitors (Atezolizumab, Avelumab, Durvalumab) and two PD-1 inhibitors (Pembrolizumab, and Nivolumab) have been approved by the U.S. Food and Drug Administration (FDA) for the treatment of melanoma, lymphoma, non-small cell lung cancer, liver cancer, bladder cancer, head and neck cancers, and kidney cancer. In addition, PD-1/PD-L1 inhibitors are being investigated in clinical trials for many other cancers, such as prostate cancer, colorectal cancer, breast cancer, ovarian cancer, pancreatic cancer, gastric cancer, and glioblastoma. Moreover, PD-1/PD-L1 inhibitors are being used in combination with various chemotherapy agents to improve their therapeutic index [1].

Currently, all the approved checkpoint inhibitors are monoclonal antibodies. Although antibody-based checkpoint inhibitors have demonstrated impressive efficacy, major limitations still exist during clinic applications, such as immune-related adverse events (irAEs) because of the breaking of immune self-tolerance in normal tissues, high cost, and immunogenic response after repeated administrations [3]. One critical disadvantage of antibody-based checkpoint inhibitors is their poor tumor penetration due to large size (150 kDa) [4, 5]. As a result, the antibodies may exert limited blockade effect within solid tumors, leading to suboptimal efficacy. Another drawback of the antibodies is their Fc-mediated activation of cytotoxic immune responses through macrophages and natural killer cells, which results in undesirable depletion of T cells in the circulation. For example, PD-1 and PD-L1 are expressed on the surface of antitumor cytotoxic T cells, and immunotherapy with anti-PD-1 antibodies was reported to lower the number of circulating T-cells in patients, thus comprising the efficacy of immunotherapy [6–8].

To address the deficiencies of antibody-based checkpoint inhibitors, there has been a growing interest in developing low-molecular-weight checkpoint inhibitors in the past few years [3, 9, 10]. However, there is an inherent challenge in discovering small-molecule drugs (Mw < 500 Da) to block immune checkpoints because of the relatively large and flat interface of the receptor/ligand interaction without well-defined pockets [3, 10]. Instead, small synthetic peptides could be promising candidates to block such receptor/ligand interactions, and a few peptides have been reported recently [11, 12]. Compared to antibodies, small synthetic peptides have several advantages, including ease of manufacture, reduced immunogenicity, better tumor penetration, and lack of Fc-mediated side effects [5, 13]. The most significant advantage of low-molecular-weight peptides is that they can efficiently penetrate into tumors and block PD-1/PD-L1 interaction not only near tumor vasculature but also distal from the vasculature. Moreover, low-molecular-weight anti-PD-L1 peptides can be easily linked to a targeting ligand or encapsulated in a nanoscale delivery system to improve their specificity to tumor cells, thus minimizing the non-specific blockade effect in other tissues expressing PD-L1. Given all of the evidence described above, peptide-based checkpoint inhibitors are considered attractive candidates for cancer immunotherapy.

Phage display biopanning is an affinity selection technology using a phage library, which contains billions of different phages, and each phage expresses a unique inserted peptide or protein sequence on the surface. Phage display biopanning resembles, in essence, the affinity selection using traditional chemical libraries, but with a much more comprehensive library including literally billions of different peptides. Phage display biopanning therefore provides a high throughout tool to identify peptide candidates against a wide variety of molecular targets including proteins, cells, and animal tissues [14]. These peptide candidates have been widely used as targeting ligands for drug delivery systems or imaging agents. Moreover, these peptide ligands can be explored as therapeutic agents, such as vaccines [15], mimotope-based immunotherapy agents [16], or inhibitors of a target protein [17].

In the present study, we developed a novel biopanning strategy and discovered anti-PD-L1 peptide inhibitors (12 aa, ~ 1.6 kDa) to block the PD-1/PD-L1 interaction. The peptides exhibit high affinity and specificity to human PD-L1 protein as well as PD-L1-positive human cancer cells MDA-MB-231 and DU-145. Molecular docking studies indicate that the CLP002 peptide specifically binds to PD-L1 at the residues where PD-L1 interacts with PD-1. The peptide also blocks the CD80/PD-L1 interaction, which may further enhance the immune response of tumor-infiltrating T cells. The CLP002 peptide restores proliferation and prevents apoptosis of T cells that are co-cultured with cancer cells. The CLP002 peptide also inhibits tumor growth and increases survival of CT26 tumor-bearing mice, suggesting that the CLP002 peptide represents a promising low-molecular-weight checkpoint inhibitor for cancer immunotherapy.

## Methods

### Cell culture

MDA-MB231, DU-145, CT26, 4T1 and Jurkat cells were purchased from ATCC. MDA-MB231 and DU-145 cells were cultured in DMEM medium with 10% Fetal Bovine Serum (FBS), 100 units/mL penicillin and 100 μg/mL streptomycin. CT26, 4 T1, and Jurkat cells were cultured in RPMI1640 medium with 10% FBS, 100 units/mL penicillin and 100 μg/mL streptomycin. All cells were grown at 37 °C in a humidified atmosphere containing 5% CO2.

### Biopanning procedure

The recombinant human PD-L1 extracellular domain (ECD) protein (cat# FCL0784B, G&P Biosciences, Santa Clara, CA) was coated on two wells of a 96-well plate. On the first well, PD-L1 was incubated with PD-1 protein, followed by incubation with the Ph.D.™-12 Phage Display Peptide Library (NEB, Ipswich, MA). The unbound phages were transferred to the second well, which was coated with PD-L1. The bounded phages were eluted from the second well and amplified. In each biopanning, approximately 10^11^ pfu phages were loaded, and eluted phages were tittered and amplified for the next round of selection.

### Blockade of the PD-1/PD-L1 interaction

Ninety-six-well plates were coated with 100 ng of PD-L1 protein (G&P Biosciences, human PD-L1 ECD, cat# FCL0784B. mouse PD-L1, cat# FCL3502B) and later blocked with 2% BSA for 2 h at room temperature. Various concentrations of peptides were loaded into the wells and incubated for 1 h at room temperature. Biotinylated PD-1 (G&P Biosciences, human PD-1 ECD, cat# FCL0761B; mouse PD-1, cat# FCL1846) was added and incubated for 1 h. Streptavidin-HRP (R&D systems) and chromogenic substrate were added into the wells. OD_450_ was then recorded and referenced to OD_540_.

### Evaluation of binding kinetics and affinity by surface Plasmon resonance (SPR)

Binding affinities of the PD-L1 specific peptides for human PD-L1 protein were determined by SPR (BI4500, Biosensing Instrument). PD-L1 protein was diluted to 10 μg/mL with sodium acetate buffer (pH 5.0, GE Healthcare, PA) and covalently coated onto a CM5 sensor chip (CM Dextran Sensor Chip, Biosensing Instrument) using the standard Amine Coupling Kit (GE Healthcare, PA). Approximately 6500 RU of PD-L1 protein were immobilized onto the chip. A second channel was used as a reference. HBS-EP+ buffer (GE Healthcare) was employed at a flow rate of 60 μL/min. A series of concentrations of each peptide (15, 30, 60, 125, 250, 500, 1000, 5000 and 10,000 nM) were prepared in HBS-EP+ running buffer to obtain the equilibrium dissociation constant (K_D_) values of the peptides. The CM5 sensor chip was regenerated with 10 mM NaOH for 20 s. The results were analyzed using the software of Bi data analysis software [11].

### Binding specificity of the anti-PD-L1 peptides towards PD-L1 overexpressing cancer cells

Binding of the peptides to PD-L1-positive cancer cells (MDA-MB-231 and DU-145) and PD-L1-negative cancer cells (MCF-7) was evaluated as we described before with modifications [18]. The cells were treated with the non-enzymatic cell dissociation solution (MP Biomedicals, Santa Ana, CA) and diluted to a density of 1 × 10^6^ cells/mL in Opti-MEM. The suspended cells were incubated with various concentrations of 5-FAM-labeled anti-PD-L1 peptides or Cy5-labeled PD-L1 antibody for 1 h at 37 °C with gentle rotation. After washing, the cells were analyzed using a FACSCalibur flow cytometer (BD Biosciences, Franklin Lakes, NJ).

### 3D tumor spheroid penetration assay

3D spheroids of MDA-MB-231 cells were generated using the Spheroid Formation Extracellular Matrix (ECM) as per the company’s protocol (Amsbio, Cambridge, MA). Briefly, 3000 tumor cells were mixed with 50 μL Spheroid Formation ECM and loaded into a Corning™ 96-well Ultra-low Attachment Treated Spheroid Microplate (Corning, Pittsburgh, PA). The plate was centrifuged at 200 g for 3 min at 4 °C. The cells were then incubated at 37 °C until the diameter reached approximately 700 μm. The Cy5-labeled CLP002 peptide and anti-PD-L1 antibody were incubated with the spheroids for 2 or 6 h. After washing, penetration of the peptide and antibody inside the tumor spheroids was determined using confocal microscopy.

### Proliferation and apoptosis assays

Proliferation and apoptosis of Jurkat T cells were assessed as described [19, 20]. Briefly, 3 × 10^4^ Jurkat T cells were cultured alone or co-cultured with 1.5 × 10^5^ DU-145 cancer cells in a 24-well plate for the proliferation assay. For the apoptosis assay, 6 × 10^4^ Jurkat T cells were cultured alone or co-cultured with 3 × 10^5^ DU-145 cancer cells in a 6-well plate. After incubation with the anti-PD-L1 peptides (5 μM) or anti-PD-L1 antibody (1 μM) for 24 h, Jurkat T cells were harvested from the supernatant. Proliferation of Jurkat cells was determined using the CellTiter-Glo luminescent cell viability assay (Promega, WI), and apoptosis of the cells were determined using the Dead Cell Apoptosis Kit with Annexin V Alexa Fluor® 488 and Propidium Iodide (Thermo-Fisher Scientific, Pittsburgh, PA) as we described before [21].

### Molecular docking of the binding of the anti-PD-L1 peptides

The crystal structure of human PD-L1 protein (PDB ID: 5C3T) and its binding residues to PD-1 were reported previously [22]. Structures of the peptides were generated using BIOVIA Draw (BIOVIA) and then aligned to the PD-L1 structure using Autodock Vina. Illustrations of the PD-L1 protein and peptide complex were generated using Pymol (Delano Scientific).

### Animal study

The animal protocol was approved by the University of Missouri-Kansas City, Institutional Animal Care and Use Committee (IACUC). Five-week old male and female Balb/c mice were purchased from Charles Rivers Laboratories (Wilmington, Massachusetts) and housed in a temperature and humidity controlled room on a 12-h light-dark cycle. Approximately 5 × 10^5^ CT26 cells were subcutaneously injected into the right flank. The mice were randomly divided into five different groups (10 mice/group, 50% female, 50% male). The mice were intraperitoneally injected with 2 mg/kg peptide daily when the tumor size reached 50–100 mm^3^. The anti-mouse PD-L1 antibody (10F.9G2, BioXcell) was administrated as a positive control at a dose of 10 mg/kg every two days. The tumor size was assessed with a caliper and calculated with the formula 0.5 × length×width^2^. ELISA kits of PD-L1 (Cat# DY1019–05), IFNγ (Cat# DY485–05), and IL-6 (Cat# DY406–05) were used to measure the expressions of PD-L1, IFNγ, and IL-6, respectively, in tumors as per the company’s instructions (R&D Systems, Minneapolis, MN).

### Immunohistochemistry (IHC) staining

Tumor tissues were fixed in 10% formalin, embedded in paraffin, sectioned, and mounted on glass slides by Truman Medical Centers Anatomic Pathology Core (Kansas City, MO). The slides were heated in Tris buffer (pH 9.0) for 45 min to recover antigen. After deparaffinization and rehydration, the sections were stained with the anti-mouse CD8 alpha antibody (Abcam, ab209775) overnight at 4 °C. The slides were incubated with a biotinylated goat anti-rabbit secondary antibody, followed by the DAB chromogen mixture. Four sections (2 male and 2 female) were imaged in each group. For each section, 3 regions were randomly selected for imaging.

### Statistical analysis

Data are expressed as the mean ± standard deviation (SD). The difference between any two groups was determined by one-way analysis of variance (ANOVA) with Tukey’s post hoc test. For the tumor volume, the difference between any two groups was determined by two-way ANOVA with Tukey’s post hoc test. *P* < 0.05 was considered statistically significant.

## Results

### Discovery of anti-PD-L1 peptides using biopanning

**Fig. 1:**
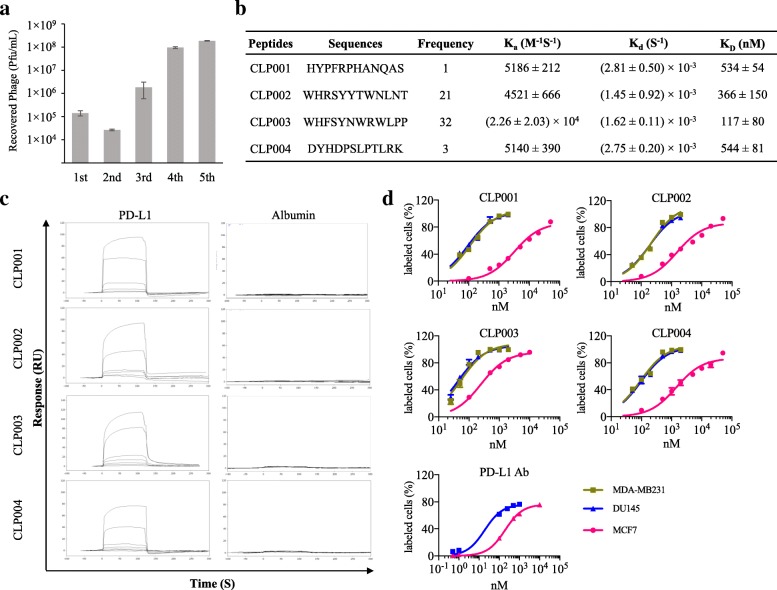
Discovery of anti-PD-L1 peptides using a novel biopanning procedure. **a** The number of recovered phages from each round of biopanning. **b** Sequences of the discovered anti-PD-L1 peptides. **c** Binding affinities of the selected peptides towards human PD-L1 protein and albumin were measured using SPR. **d** Binding curves of the peptides on PD-L1-positive human cancer cells (MDA-MB-231 and DU-145) and PD-L1 deficient human cancer cells MCF-7. Binding curves of the anti-PD-L1 antibody were measured on DU-145 and MCF-7 cells. Results are represented as the mean ± SD (*n* = 3)

The aim of this study is to discover small peptides not only specifically bind to PD-L1 but also block the interaction between PD-L1 and PD-1. We developed a novel biopanning strategy to select peptides that specifically bind to PD-L1. As Fig. 1a shows, after five rounds of biopanning, the number of eluted phages increased dramatically, indicating significant enrichment of PD-L1-specific phages in the elution. Totally 57 single phage colonies were randomly selected for sequencing, and 4 peptide sequences were discovered (Fig. 1b). The CLP002 peptide and CLP003 peptide have 21 and 32 repeats, respectively, while the CLP001 peptide and CLP004 peptide have 1 and 3 repeats, respectively.

### Binding affinity and specificity of the peptides to PD-L1

Binding affinities of the discovered peptides against the recombinant human PD-L1 ECD protein were evaluated using SPR. The PD-L1 protein was immobilized on a CM5 golden chip by the direct amine coupling method. As illustrated in Fig. 1b, the K_D_ values of CLP001, CLP002, CLP003 and CLP004 for human PD-L1 were 534, 366, 117, and 544 nM, respectively. The peptides are considerable competitive against the PD-1/PD-L1 interaction (K_D_ = ~ 4 μM) [23]. Even though the binding affinity of peptides are generally lower than that of antibodies, the very high affinity of antibodies may result in on-target, off-tumor toxicity in healthy tissues that express low levels of PD-L1. In a recent study, scientists constructed several chimeric antigen receptor (CAR) T cells with different affinities to ICAM-1. CAR-T cells with micromolar affinity to ICAM-1 showed better antitumor efficacy and safety than CAR-T cells with nanomolar affinity. CAR-T cells with nanomolar affinity lyse healthy cells that express a low level of ICAM-1. By contrast, CAR-T cells with micromolar affinity only attack tumor cells with high levels of ICAM-1 but not healthy cells with low levels of ICAM-1, leading to less toxicity [24].

Having shown the high affinity of the peptides to PD-L1, we next examined the specificity of these peptides. We first measured nonspecific binding of the peptides against bovine serum albumin (BSA) using SPR. As revealed in Fig. 1c, the response curves of BSA to the peptides did not change with gradient concentrations of the peptides, indicating negligible binding between the peptides and BSA. By contrast, the response curves of the peptides to the PD-L1 protein were correlated with the peptide concentrations. We further examined their specificity to PD-L1-positive human cancer cells DU-145. MCF-7 human cancer cells are PD-L1 deficient and used as a negative control in this study [25, 26]. As Fig. 1d revealed, all peptides and PD-L1 antibody (29E.2A3, BioXcell, West Lebanon, NH) exhibited high binding affinity to PD-L1-positive cancer cells (DU-145) but low affinity to PD-L1-deficient human cancer cells MCF-7. These results clearly suggest that the anti-PD-L1 peptides specifically bind to recombinant human PD-L1 protein as well as PD-L1 overexpressing human cancer cells.

### Blockade of the PD-1/PD-L1 interaction

**Fig. 2:**
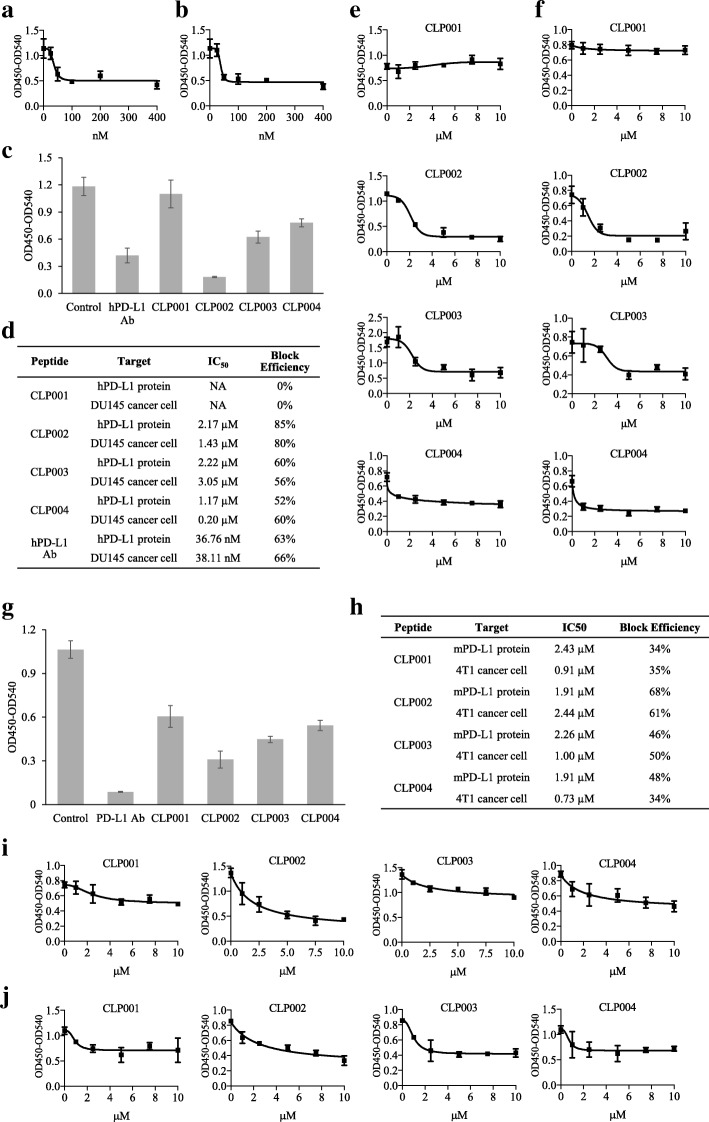
Blockade of the PD-1/PD-L1 interaction by the anti-PD-L1 peptides and antibody. **a** Blocking profile of the anti-human PD-L1 antibody (R&D, AF156) against human PD-L1 protein. **b** Blocking profile of the anti-human PD-L1 antibody (R&D, AF156) against DU-145 cells. **c** Blocking efficiency of the anti-PD-L1 peptides (10 μM) and the anti-human PD-L1 antibody (1 μM) against human PD-L1 protein. **d** IC_50_ and blocking efficiency of the peptides and antibody against human PD-L1 protein and human cancer cell line DU-145. **e** Blocking profiles of the peptides against human PD-L1 protein. **f** Blocking profiles of the peptides against DU-145 cells. **g** Blocking efficiency of the peptides and an anti-mouse PD-L1 antibody (BioXcell, 10F.9G2) at 10 μM against a mouse PD-L1 protein. **h** IC_50_ and blocking efficiency of the peptides against mouse PD-L1 protein and mouse cancer cell line 4 T1. **i** Blocking profiles of the peptides against mouse PD-L1 protein. **j** Blocking profiles of the peptides against mouse cancer cell line 4 T1. Results are represented as the mean ± SD (n = 3)

We next determined whether the anti-PD-L1 peptides block the human PD-1/PD-L1 interaction. An anti-human PD-L1 antibody (R&D Systems, cat# AF156) was used as a positive control to calibrate this assay. As shown in Fig. 2a, the anti-human PD-L1 antibody blocked the PD-1/PD-L1 interaction with a half maximal inhibitory concentration (IC_50_) of 36.76 nM, which is consistent with the report from the company. IC_50_ of the antibody against PD-L1 overexpressing DU-145 cancer cells is 38.11 nM, which is comparable to the blocking effect on PD-L1 protein (Fig. 2b). We next examined the blocking efficiency of the anti-PD-L1 peptides at 10 μM (Fig. 2c). CLP002 showed the highest blocking efficiency, whereas CLP001 did not block the PD-1/PD-L1 interaction. We also determined IC_50_ of each peptide using recombinant human PD-L1 protein and DU-145 cancer cells. As revealed in Fig. 2d-f, CLP002 exerted the best blocking effect (85%) with an IC_50_ of 2.17 μM, when the plate was coated with the human PD-L1 protein. The blocking effect was 80% with an IC_50_ of 1.43 μM, when the plate was coated with DU-145 cells. IC_50_ of the CLP003 peptide was 2.22 μM with 60% blocking efficiency against the human PD-L1 protein, and the IC_50_ was 3.05 μM with a 56% blocking efficiency against DU-145 cancer cells.

Blocking efficiencies of the peptides against mouse PD-1/PD-L1 interaction was also evaluated because we will evaluate their anti-tumor activity in mice implanted with murine cancer cells. As shown in Fig. 2g, blocking efficiencies of the peptides and an anti-mouse PD-L1 antibody (BioXcell, 10F.9G2) were compared at 10 μM. CLP002 blocked 71% of the mouse PD-1/PD-L1 interactions, while CLP003 blocked approximately 46% of the interactions. By contrast, the blocking efficiency of the anti-mouse PD-L1 antibody was 92%. This is because CLP002 and CLP003 were discovered against human PD-L1 protein and therefore may have less binding affinity to mouse PD-L1 or less overlap with the mouse PD-1/PD-L1 interaction residues. We also determined the IC_50_ values of the peptides to block the mouse PD-1/PD-L1 interaction. As revealed in Fig. 2h to j, the IC_50_ of CLP002 was 1.91 μM with a 68% blocking efficiency, while the IC_50_ of CLP003 was 2.26 μM with a 46% blocking efficiency. In summary, the CLP002 peptide displayed the highest blocking efficiency against the PD-1/PD-L1 interaction and therefore was selected as the best PD-L1-specific peptide for subsequent activity studies.

It has been reported that PD-L1 binds to CD80 with a moderate binding affinity, and the CD80/PD-L1 interaction interface is partially overlapped with PD-1/PD-L1 and CD80/CTLA4 interfaces. The CD80/PD-L1 interaction specifically restrains T cell activation, and blockade of the interaction could enhance the anti-tumor activity of the T cells [27]. For example, Durvalumab is a FDA-approved anti-PD-L1 antibody that blocks not only the PD-1/PD-L1 but also the CD80/PD-L1 interaction [28]. We, therefore, investigated whether the anti-PD-L1 peptides block the CD80/PD-L1 interaction. As revealed in Additional file 1: Figure S1, we observed approximately 17, 48, 48 and 27% blocking efficiency of the peptides CLP001, CLP002, CLP003, and CLP004 at 10 μM, respectively. The IC_50_ values for the peptides CLP002 and CLP003 are 2.45 μM and 1.62 μM, respectively. The data suggest that the anti-PD-L1 peptides block PD-1/PD-L1 and CD80/PD-L1 interactions simultaneously, leading to enhanced anti-tumor activity of the T cells.

### Molecular docking for the peptide/PD-L1 interaction

**Fig. 3:**
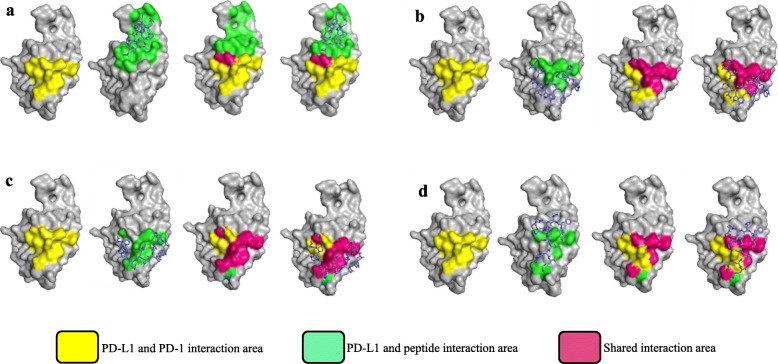
Molecular docking for the interaction between the anti-PD-L1 peptides and human PD-L1 protein (PDB ID: 5C3T). **a** Modeling of the interaction between CLP001 and PD-L1. **b** Modeling of the interaction between CLP002 and PD-L1. **c** Modeling of the interaction between CLP003 and PD-L1. **d** Modeling of the interaction between CLP004 and PD-L1. The PD-L1 residues responsible for peptide binding are highlighted in green. The binding residues for human PD-1 protein is highlighted in yellow. The overlapping PD-L1 residues for binding both anti-PD-L1 peptide and PD-1 protein are highlighted in pink

We performed molecular docking studies to simulate the interactions between the anti-PD-L1 peptides and the human PD-L1 extracellular domain protein (PDB ID# 5C3T) using Autodock Vina integrated into PyRx [29]. Illustrations of the PD-L1/peptide complexes were generated using Pymol (Fig. 3). The PD-L1 residues responsible for the PD-1/PD-L1 interaction were previously reported and highlighted in yellow [22]. The binding residues of CLP002 and CLP003 on PD-L1 are highly overlapped with that of PD-1 (Fig. 3b and Fig. 3c). As illustrated in Fig. 3a, the CLP001 peptide does not bind to the PD-1/PD-L1 interaction residues, which explains the fact that the CLP001 peptide binds to PD-L1 but does not block the PD-L1/PD-1 interaction (Fig. 2d-e). Similarly, there is only a small overlap between the CLP004/PD-L1 binding area and the PD-L1/PD-1 interaction residues (Fig. 3d). This is also in accordance with the poor blocking efficacy of the CLP004 peptide in Fig. 2.

### CLP002 restores T cell proliferation and prevents T cell apoptosis in the presence of PD-L1-overexpressing cancer cells

In the tumor microenvironment, PD-L1-overexpressing tumor cells inhibit T cell activation and promote T cell apoptosis, leading to exhausted phenotype and impaired effector function of the T cells [20]. The PD-1/PD-L1 interaction also suppresses T cell proliferation and inhibits the secretion of inflammatory cytokines [30]. We, therefore, co-cultured Jurkat T cells with PD-L1-overexpressing DU-145 cancer cells to investigate whether the anti-PD-L1 peptides reverse the inhibitory effect of DU-145 cancer cells on Jurkat T cells.

**Fig. 4:**
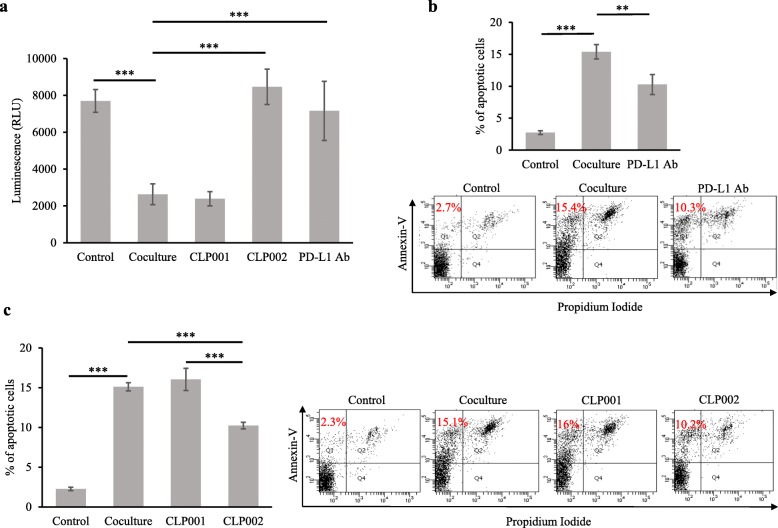
The CLP002 peptide restores T cell proliferation and prevents T cell apoptosis. Jurkat T cells were co-cultured with DU-145 cells and then incubated with the anti-PD-L1 peptides or antibody for 24 h. The CLP002 peptide and antibody restore Jurkat T cell proliferation (**a**) and reduces Jurkat T cell apoptosis (**b**-**c**) in the presence of PD-L1 overexpressing DU-145 cells. Results are represented as the mean ± SD (n = 3). (** *p* < 0.01; *** *p* < 0.001)

As revealed in Fig. 4a, DU-145 cells significantly inhibited T cell proliferation through the PD-1/PD-L1 interaction. Treatment of the co-cultured cells with the CLP002 peptide restored Jurkat T cell proliferation, which is consistent with previous reports [19, 31–33]. For example, Freeman et al. reported that human PD-L1 protein suppressed the proliferation of T cells in a dose-dependent manner. By contrast, the PD-L1 protein did not inhibit the proliferation of PD-1 knockout T cells, suggesting that the inhibitory effect of DU-145 cells is mediated by the PD-1/PD-L1 interaction [19]. In another study, PD-L1 expression on myeloid-derived suppressor cells (MDSCs) was found selectively upregulated by hypoxia-inducible factor-1 α (HIF-1α) under hypoxia, leading to suppression of T cells. Using HIF-1α or PD-L1 inhibitors reversed MDSC-mediated T cell suppression under hypoxia [31].

We also treated the co-cultured cells with the CLP001 peptide but did not observe the same effect on T cells (Fig. 4a). This is in agreement with our finding that the CLP001 peptide is not able to block the human PD-1/PD-L1 interaction (Fig. 2). These results further proved that PD-L1-overexpressing cancer cells inhibit the proliferation of Jurkat cells through the PD-1/PD-L1 interaction.

We next investigated the effect of the anti-PD-L1 peptides and anti-PD-L1 antibody on the apoptosis of Jurkat cells in the presence of DU-145 cells. As shown in (Fig. 4b and 4c) the apoptosis of Jurkat cells increased from 2.3 to 15.1%, when the cells were co-cultured with DU-145 cells. Apoptosis was effectively inhibited to 10.2 and 10.3% when the co-cultured cells were treated with the CLP002 peptide or anti PD-L1 antibody but not the CLP001 peptide. This result is in agreement with the proliferation assay (Fig. 4a) and a previous report, which concluded that PD-L1 overexpression on tumor cells promote T cell apoptosis [20]. In this report, the investigators observed increased apoptosis of cytotoxic T lymphocytes after incubation with melanoma cancer cells. The authors however did not observe apoptosis of the immune cells, when PD-L1 was knockout in melanoma tumor cells. In addition, tumor-promoted T cell apoptosis was significantly reduced by incubating with an anti-PD-1 antibody [20].

### Comparison of tumor penetration of the anti-PD-L1 peptide CLP002 and anti-PD-L1 antibody

**Fig. 5:**
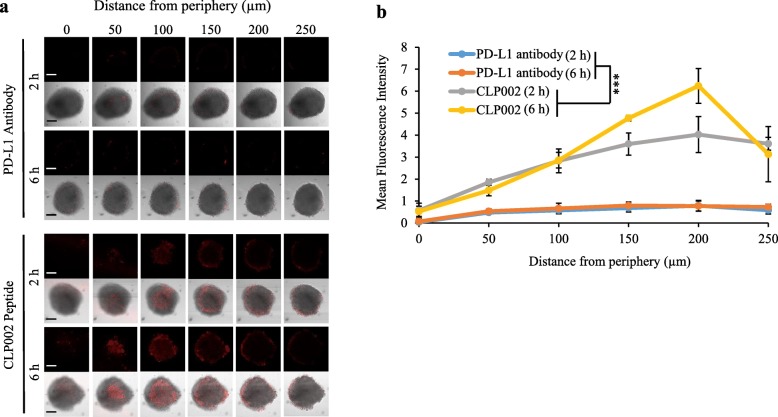
3D spheroid penetration of the CLP002 peptide and anti-PD-L1 antibody. 3D tumor spheroids of MDA-MB-231 cells were generated to compare the tumor penetration capability of the CLP002 peptide and the anti-PD-L1 antibody (BioXcell, 29E.2A3). Cy5-labeled peptide and antibody were incubated with the tumor spheroids (~ 700 μm in diameter) for 2 and 6 h, followed by confocal microscopy analysis to evaluate tumor penetration. **a** Representative Z-stacked confocal images of the spheroids with a z-step of 50 μm. The scale bar represents 200 μm. **b** The depth of penetration is quantified by mean fluorescence intensity. Results are represented as the mean ± SD (n = 3)

We hypothesize that low-molecular-weight peptides have better tumor penetration than antibodies, which may lead to improved therapeutic efficacy. A 3D tumor spheroid model of MDA-MB-231 cells was developed to compare tumor penetration of the CLP002 peptide and the anti-PD-L1 antibody (29E.2A3, BioXcell, West Lebanon, NH). Cy5-labeled peptide and antibody were incubated with the tumor spheroids (~ 700 μm in diameter) for 2 and 6 h, followed by confocal microscopy analysis to evaluate tumor penetration. As illustrated in Fig. 5a-b, the CLP002 peptide exhibited better tumor penetration than the antibody. Fluorescence of the Cy5-labeled CLP002 peptide was detected as deep as approximately 250 μm from the periphery of the spheroids. By contrast, Cy5-labeled antibody was only detected on the periphery of the spheroids, suggesting very limited tumor penetration.

### Antitumor activity of the anti-PD-L1 peptides

**Fig. 6:**
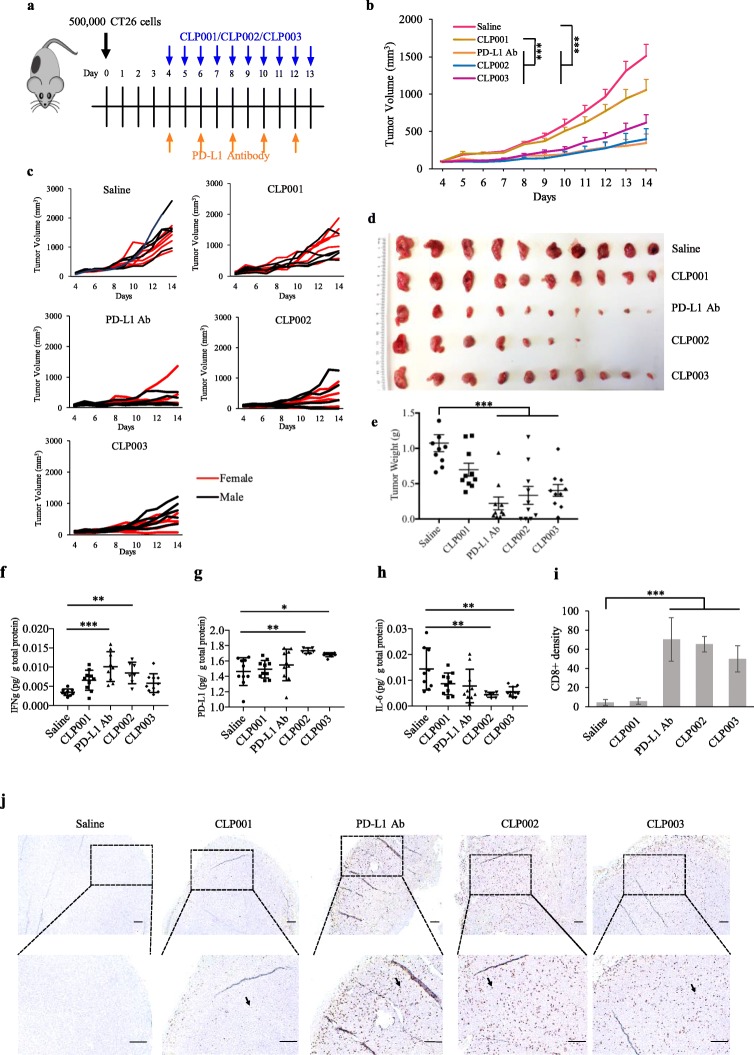
Anti-tumor activity of the anti-PD-L1 peptides and antibody. **a** CT26 tumor-bearing Balb/C mice (*n* = 10, 5 male and 5 female) were intraperitoneally injected with the anti-PD-L1 peptides (2 mg/Kg) daily for a total of 10 injections and the anti-mouse PD-L1 antibody (10 mg/Kg) every other day for a total of 5 injections. **b** Tumor volume measured over time. Tumor volume results were represented as the mean ± SE (n = 10). **c** Tumor growth curves of individual mice in each group. Image **d** and weight **e** of tumors harvested at day 14. The results were represented as the mean ± SD (n = 10). The expressions of IFNγ **f**, PD-L1 **g** and IL-6 **h** in harvested tumors were measured using ELISA. **i** The numbers of CD8^+^ T cells in each specimen were quantitated after immunohistochemical staining. Results were represented as the mean ± SD (*n* = 4). **j** Representative images of tumor specimen stained with anti-CD8 antibody. The scale bar represents 200 μm. (* *p* < 0.05; ** p < 0.01; *** p < 0.001)

We evaluated the antitumor activity of the anti-PD-L1 peptides using the CT26 colorectal tumor-bearing mouse model (Fig. 6a), which has been widely used to evaluate the activity of PD-1/PD-L1 inhibitors [11, 34]. Once the average tumor volume reached 50–100 mm^3^, the peptides (2 mg/kg) were administrated intraperitoneally daily, as described in a previous study [11]. The anti-mouse PD-L1 antibody (BioXcell, 10F.9G2) was administered intraperitoneally every other day at 10 mg/kg as reported [35]. As Fig. 6b to d showed, CLP002, CLP003, and the antibody effectively suppressed tumor growth. As shown in Fig. 6e, the tumor weights of the PD-L1 antibody, CLP002 and CLP003 group were significantly smaller than the saline group. In general, CLP002 exerted a better tumor inhibitory effect than CLP003, which was similar to the antibody. It is noteworthy to mention that the peptides were screened against the human PD-L1 protein, which, in fact, would compromise the anti-tumor activity of the peptides in a mouse model. We are, therefore, cautiously optimistic with the antitumor activity of the peptides in human cancer cells.

Next, we evaluated the expressions of PD-L1 and cytokines associated with anti-tumor immune responses. IFNγ is a multifunctional cytokine secreted by activated T cells. It was generally thought that the therapeutic effect of cytotoxic T cells is mainly mediated by the secretion of IFNγ [36, 37]. However, IFNγ may also upregulate PD-L1 expression on cancer cells to escape T cell-mediated immune response [38]. Consistent with a previous report [36], we observed upregulated IFNγ and PD-L1 levels in the tumors after the treatment with the anti-PD-L1 antibody and the peptides (Fig. 6f and g). Because IFNγ induces the expression of PD-L1 on tumor cells, we also observed slightly higher PD-L1 levels of the treated tumor tissue. As Mandai et al. reported, IFNγ initially triggers immune response through the T cell activation. PD-L1 expression was also elevated by secreted IFNγ, which facilitates the escape of tumors from T cell-mediated immune response [39].

IL-6 is often upregulated along with tumor growth. For example, prominent IL-6 expression was detected in pancreatic tumor microenvironment, which is critical for tumor progression [40]. The IL-6/STAT3 pathway facilitates the expansion of immunosuppressive cells or changes the balance of T cell subsets, such as T regulatory cells and MDSCs, which promote tumor growth. Blockade of IL-6 with an antibody inhibits tumor growth and enhances survival in mice bearing aggressive pancreatic cancer cells [40]. In a clinical study, the expression of IL-6 in the blood was found to be reduced in cancer patients who received the treatment of the anti-PD-L1 antibody MPDL3280A [41]. In agreement with these reports, we observed a decreased expression of IL-6 in the tumor tissues after the treatment with CLP002 and CLP003 (Fig. 6h).

CD8^+^ cytotoxic T lymphocytes play critical roles in cancer immunotherapy using checkpoint inhibitors. For example, Tumeh et al. analyzed tumor specimen from patients receiving anti-PD-1 antibody therapy and observed proliferation of intratumoral CD8^+^ T cells, which was correlated with the therapeutic outcome of the immunotherapy. Patients responding to the therapy showed higher density of CD8^+^ T cells [37]. We therefore performed immunohistochemical staining for CD8^+^ T cells in the tumor tissues. In agreement with the previous report, both the antibody and the anti-PD-L1 peptides (CLP002 and CLP003) significantly increased the density of CD8^+^ T cells in tumor tissues (Fig. 6i and j). In addition, we observed penetration of CD8^+^ T cells into the tumor tissue of CLP002-treated mice. By contrast, CD8^+^ T cells were mainly detected on the periphery of the tumors in PD-L1 antibody treated mice. This could be due to the better tumor penetration of the peptides (as shown in Fig. 5), which promote the infiltration or proliferation of CD8^+^ T cells.

**Fig. 7:**
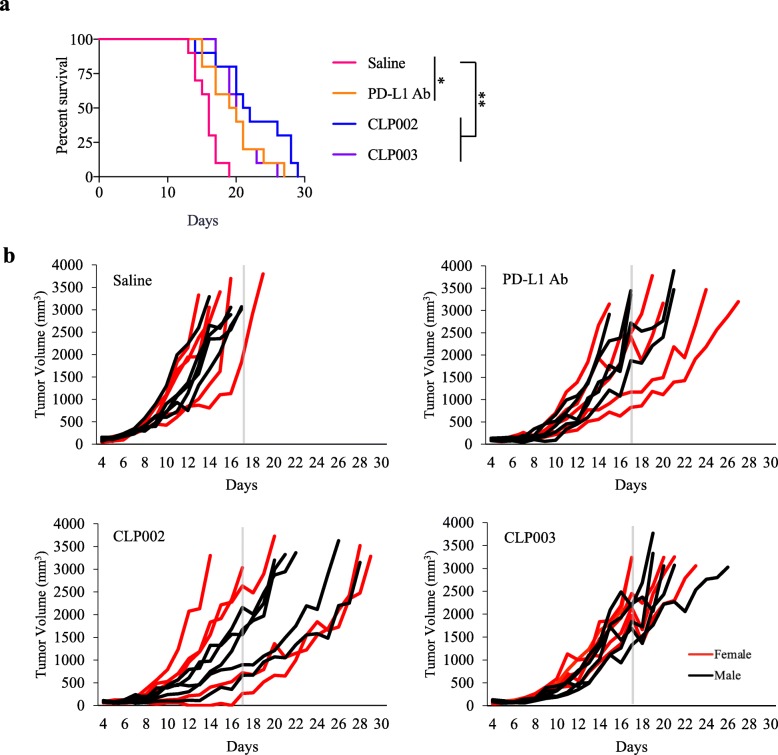
Survival curves of the mice treated with the Anti-PD-L1 peptides and PD-L1 antibody. CT26 tumor-bearing Balb/C mice (n = 10, 5 male and 5 female) were intraperitoneally injected with the anti-PD-L1 peptides (2 mg/Kg) daily and the anti-mouse PD-L1 antibody (10 mg/Kg) every other day from day 4 to day 17. **a** Survival curves. GraphPad Prism 7 software (San Diego, CA) was used for statistical analysis. Comparison of two survival curves were conducted using the Gehan-Breslow-Wilcoxon test. **b** Tumor growth curves of individual mice in each group

For the survival study, mice were intraperitoneally injected with the CLP002 and CLP003 peptides daily or the anti-mouse PD-L1 antibody every other day from day 4 to day 17 (Fig. 7a). CLP002 inhibited tumor growth and improved the survival of tumor-bearing mice compared with control animals. CLP003 and the PD-L1 antibody exerted a similar effect and modestly improved the survival of tumor-bearing mice. As shown in Fig. 7b, 90% of the mice in the saline group had died by day 17. By contrast, only 20% of the mice in the CLP002 group were dead by day 17. Eight mice showed a response to the CLP002 treatment, which is better than PD-L1 antibody treated mice (60% response). For CLP003, though only one mouse died on day 17, other mice experienced a rapid tumor progression.

## Discussion

Synthetic peptides have been widely used as targeting moieties and therapeutic reagents to treat various diseases, but the applications of peptides as checkpoint inhibitors remain to be investigated [42]. During the last decade, peptide therapeutics has attracted great attention in pharmaceutical research and development. The FDA has so far approved more than 60 peptide therapeutics, among which 22 were recently approved in the period from 2011 to 2017. In addition, there are currently more than 600 peptide therapeutics in clinical and preclinical trials [43, 44]. The number of peptide therapeutics in the clinic will undoubtedly continue to increase in the coming years. Compared to small molecules which often trigger side effects by toxic metabolites or nonspecific accumulation in the body, peptides can be metabolized to amino acids in the body and have a rare incidence of side effects [45]. On the other hand, peptides are much smaller than large proteins and antibodies. Low-molecular-weight peptides therefore fulfills the need for mid-size therapeutic agents with high efficacy and low toxicity [43].

Particularly, peptides are appealing candidates for targeting protein/protein interactions, which is a difficult task for traditional small molecules [46]. Compared to small molecules, peptides are bigger and therefore can cover a significant portion of the target interface [43]. For example, the contact surface of a typical protein/protein interaction is approximately 1500–3000 A^2^, but the contact area for protein-small molecules is only 300–1000 A^2^ [47]. In addition, most protein/protein interaction interfaces are relatively featureless and lack well-defined pockets for small molecule ligands [48]. Peptides thus are more efficient to block protein/protein interactions, such as the interactions between checkpoints and their receptors. Moreover, prediction of human doses of peptides through allometric scaling is more straightforward than that of small molecules [49].

The Nobel Prize in Chemistry 2018 was awarded to scientists for “the phage display of peptides and antibodies,” indicating the great promise of the affinity selection technology. However, the traditional biopanning procedure with a peptide-expressing phage display library only screens peptides as ligands for a target protein. The peptide ligands bind to the protein but may not be able to bind specific residues of the protein. Thus, the traditional biopanning procedure cannot be used to screen peptides as inhibitors to block a protein-protein interaction. Herein, we developed a novel biopanning procedure to discover peptide inhibitors that bind to specific residues of a target protein and subsequently block the protein’s interaction with its receptor. Using the new procedure, we discovered four peptides, and all of them exhibited high and specific affinity to PD-L1. Particularly, the peptides CLP002 and CLP003 exhibited high blocking efficacy against the PD-1/PD-L1 interaction on recombinant PD-L1 protein and PD-L1-expressing tumor cells (Figs. 3 and 4). The docking results confirmed that both CLP002 and CLP003 peptides blockade the PD-1/PD-L1 interaction. The binding site of CLP002 is extensively overlapped with the PD-1/PD-L1 binding residues, which explains why CLP002 competes with PD-1 for the PD-L1 binding and blocks the interaction. These results demonstrate the feasibility of the biopanning procedure in discovering peptide-based checkpoint inhibitors.

Compared to antibodies, low-molecular-weight peptides have several advantages, such as reduced immunogenicity, ease of manufacture, better tumor penetration, and lack of Fc-mediated side effects [5, 13]. The most significant advantage of low-molecular-weight peptides is the efficient tumor penetration and blocking of PD-1/PD-L1 interaction even distal from the vasculature. For the first time, we demonstrated higher tumor penetration of a low-molecular-weight peptide compared to its counterpart antibody in a 3D tumor spheroid model (Fig. 5). Incomplete penetration into the tumor is a major limitation for macromolecular therapeutics, such as antibodies. The penetration rate of a macromolecule is highly dependent on its molecular size and binding affinity to tumor cells. While tumor penetration is inversely correlated to macromolecule’s molecular size, the correlation between tumor penetration and macromolecule’s affinity is complicated. It is generally believed that increasing the affinity of a macromolecule leads to enhanced tumor retention. However, the very high binding affinity of an antibody prevent its tumor penetration because of the “binding site barrier” effect. The antibody strongly binds to tumor cell surface but cannot diffuse into the tumor microenvironment [50, 51]. Compared to antibodies, peptides pertain much smaller size and relatively lower binding affinity, which lead to better tumor spheroid penetration.

In the animal study, we used an anti-mouse PD-L1 antibody (10F.9G2), which has been widely used as the PD-L1 inhibitor in various animal studies, as a positive control [35]. Consistent with a previous report [34], mice treated with the PD-L1 antibody showed a slower tumor growth rate. Similarly, the CLP002 peptide also inhibited tumor growth in the mice (Fig. 6). Considering the fact that the CLP002 peptide was screened against human PD-L1 protein, which only exhibits 76% sequence identity with mouse PD-L1 protein, the antitumor activity of the CLP002 peptide in a mouse model implanted with mouse tumor cells is remarkable. The peptide inhibitor also prolonged the survival of the tumor-bearing mice compared to either the saline-treated mice or the antibody-treated mice (Fig. 7). It is worthy to mention that the dose of the anti-PD-L1 peptides (2 mg/Kg daily) is lower than that of the anti-mouse PD-L1 antibody (10 mg/Kg every other day). We therefore cautiously believe that the peptide is more efficient than the antibody in inducing anti-tumor immune response.

## Conclusions

In conclusion, we developed a novel biopanning procedure and discovered several anti-human PD-L1 peptides. Particularly, the CLP002 peptide specifically binds to PD-L1 with high affinity and blocks the PD-1/PD-L1 interaction on tumor cells. The peptide exhibits better tumor penetration compared to anti-PD-L1 antibody. The peptide also inhibits tumor growth and increases survival of CT26 tumor-bearing mice. Taken together, our evidence suggests that the CLP002 peptide is a promising low-molecular-weight inhibitor for cancer immunotherapy. Moreover, low-molecular-weight anti-PD-L1 peptides can be easily linked to a targeting ligand or encapsulated in a nanoscale delivery system to improve their accumulation in the tumor microenvironment, thus minimizing the non-specific blockade effect in other tissues expressing PD-L1.

## Additional file

Additional file 1: **Figure S1.** Blockade of the PD-L1/CD80 interaction by anti-PD-L1 peptides and antibody. (a) Blocking efficiency of the anti-PD-L1 peptides and anti-human PD-L1 antibody at 10 μM against the human PD-L1/CD80 interaction. IC_50_ and blocking efficiency of CLP002 (b) and CLP003 (c) against the human PD-L1/CD80 interaction. Results are represented as the mean ± SD (*n* = 3).

## Data Availability

All data generated or analyzed during this study are included in this published article and its supplementary information files.

## Abbreviations

3D: Three-dimensional
BSA: Bovine serum albumin
CAR: Chimeric antigen receptor
ECD: Extracellular domain
ECM: Extracellular matrix
ELISA: Enzyme-linked immunosorbent assay
FBS: Fetal bovine serum
FDA: Food and Drug Administration
IC_50_: Half maximal inhibitory concentration
IHC: Immunohistochemistry
irAEs: Immune-related adverse events
K_D_: The equilibrium dissociation constant
MDSCs: Myeloid-derived suppressor cells
PD-1: Programmed cell death protein 1
PD-L1: Programmed death-ligand 1
SPR: Surface Plasmon Resonance
