# Supplementary figures and images for: Discovery of low-molecular weight anti-PD-L1 peptides for cancer immunotherapy
Source: J Immunother Cancer. 2019 Oct 22;7:270. doi: 10.1186/s40425-019-0705-y (PMC6805442; doi:10.1186/s40425-019-0705-y)

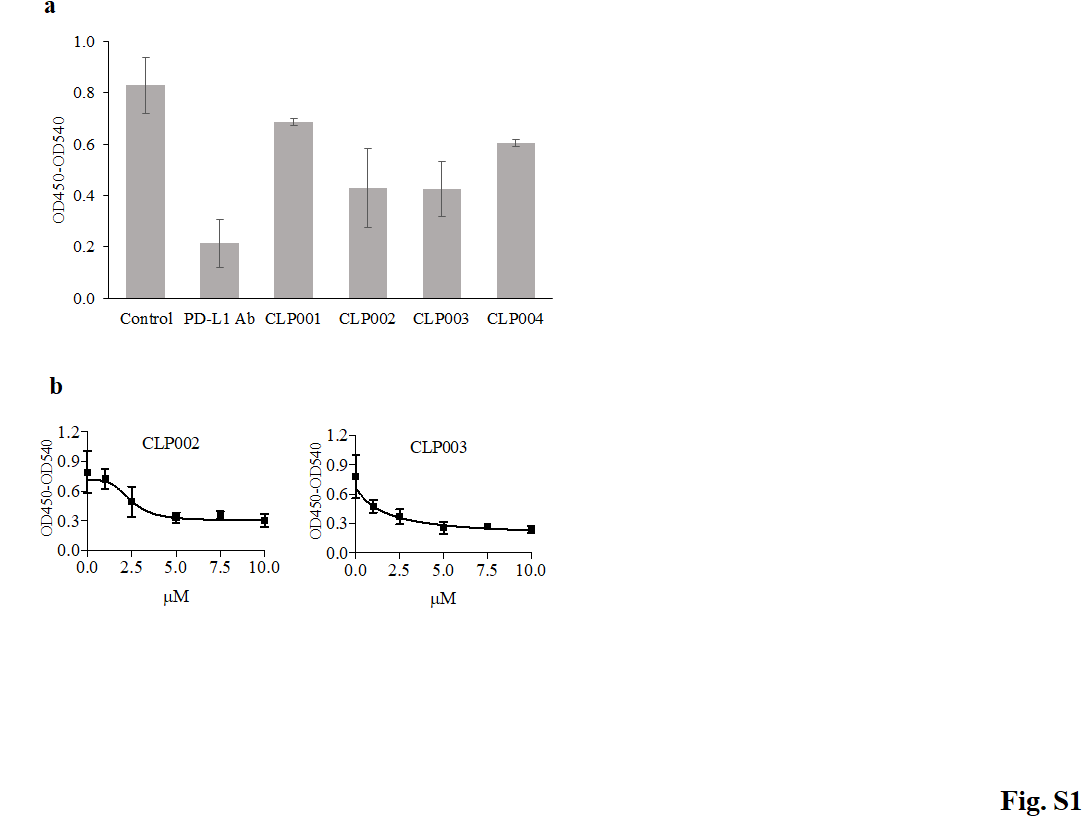

Supplement: Supplementary file 1 — Figure S1. Blockade of the PD-L1/CD80 interaction by anti-PD-L1 peptides and antibody. (a) Blocking efficiency of the anti-PD-L1 peptides and anti-human PD-L1 antibody at 10 μM against the human PD-L1/CD80 interaction. IC50 and blocking efficiency of CLP002 (b) and CLP003 (c) against the human PD-L1/CD80 interaction. Results are represented as the mean ± SD (n = 3). [file 40425_2019_705_MOESM1_ESM.png]
